# Is my wound infected? A study on the use of hyperspectral imaging to assess wound infection

**DOI:** 10.3389/fmed.2023.1165281

**Published:** 2023-08-24

**Authors:** Jose L. Ramirez-GarciaLuna, Mario A. Martinez-Jimenez, Robert D. J. Fraser, Robert Bartlett, Amy Lorincz, Zheng Liu, Gennadi Saiko, Gregory K. Berry

**Affiliations:** ^1^Department of Surgery, McGill University Health Centre, Montreal, QC, Canada; ^2^Division of Surgery, Hospital Central “Dr. Ignacio Morones Prieto”, San Luis Potosí, Mexico; ^3^Swift Medical, Toronto, ON, Canada; ^4^Arthur Labatt School of Nursing, Northwestern University, London, ON, Canada; ^5^Vope Medical, Montreal, QC, Canada; ^6^Department of Physics, Toronto Metropolitan University, Toronto, ON, Canada

**Keywords:** wounds, inflammation, infection, fluorescence, thermography, hyperspectral imaging, point-of-care, bacteria

## Abstract

**Introduction:**

Clinical signs and symptoms (CSS) of infection are a standard part of wound care, yet they can have low specificity and sensitivity, which can further vary due to clinician knowledge, experience, and education. Wound photography is becoming more widely adopted to support wound care. Thermography has been studied in the medical literature to assess signs of perfusion and inflammation for decades. Bacterial fluorescence has recently emerged as a valuable tool to detect a high bacterial load within wounds. Combining these modalities offers a potential objective screening tool for wound infection.

**Methods:**

A multi-center prospective study of 66 outpatient wound care patients used hyperspectral imaging to collect visible light, thermography, and bacterial fluorescence images. Wounds were assessed and screened using the International Wound Infection Institute (IWII) checklist for CSS of infection. Principal component analysis was performed on the images to identify wounds presenting as infected, inflamed, or non-infected.

**Results:**

The model could accurately predict all three wound classes (infected, inflamed, and non-infected) with an accuracy of 74%. They performed best on infected wounds (100% sensitivity and 91% specificity) compared to non-inflamed (sensitivity 94%, specificity 70%) and inflamed wounds (85% sensitivity, 77% specificity).

**Discussion:**

Combining multiple imaging modalities enables the application of models to improve wound assessment. Infection detection by CSS is vulnerable to subjective interpretation and variability based on clinicians' education and skills. Enabling clinicians to use point-of-care hyperspectral imaging may allow earlier infection detection and intervention, possibly preventing delays in wound healing and minimizing adverse events.

## 1. Introduction

Wound healing is achieved through the interplay of three key components: a pool of precursor cells that can proliferate and differentiate into fibroblasts and keratinocytes; neo-angiogenesis to restore the blood flow to the injury and provide nutrients and cells to the wound; and a competent immune system capable of mounting a controlled inflammatory response (1). When these components fail, wound healing stalls and a chronic non-healing wound ensues. Chronic wounds are characterized by being arrested in the progression of the wound healing phases, specifically in the inflammatory phase (2). Infections are a common trigger for developing chronic wounds and complicating the healing of those wounds that are already arrested in the inflammatory phase (3). Because the skin and wounds are non-sterile environments, it is widely accepted that wound infections occur on a spectrum that goes from contamination, colonization, local infection, and spreading infection to systemic infection (4). Thus, a common challenge for clinicians is to differentiate between contaminated and colonized wounds and wounds with subtle local infections to offer timely treatment before the infection becomes a more significant problem. Unfortunately, because clinical inspection alone has demonstrated an accuracy below 60% in identifying infected wounds (5, 6), there is a pressing need to identify diagnostic adjuncts that can help achieve better yields.

Traditionally, a microbiological assessment of the wound and peri-wound areas is used to rule out the presence of infections. However, cultures, molecular techniques, and other mainstream diagnostic results take time and are sometimes inaccessible and expensive (7). Infrared thermography (IRT) has shown promise as a tool to help diagnose inflammation and infection in wounds and skin disorders (8), as IRT heat signals have shown a high degree of correlation with inflammatory skin changes and deep, infectious processes (9).

Nonetheless, while thermal changes indicate inflammation as a proxy for infection, these changes cannot be used for diagnosing the presence of an infectious process. Another point-of-care technology that has demonstrated great potential for identifying subtle infectious processes is the use of violet light to elicit bacterial fluorescence (BF) in wounds (10). When wound bioloads <10^4^-10^5^ bacteria are present in wounds, BF can be used to identify their presence as either a red signal for porphyrin-producing organisms or a cyan one for those bacteria that produce pyoverdine pigments, with an accuracy of approximately 70% (11). However, it must be noted that BF can only identify bacteria present on the surface of wounds as this imaging technology only penetrates <1.5 mm into tissues (12), thus missing any deeper bacterial contamination or infections due to other agents, such as fungi. Therefore, despite the promising results that these technologies have demonstrated for assessing the presence of wound infections, their use alone has significant shortcomings, and the combined use of IRT and BF has not been explored.

The Swift Ray 1 (Swift Medical, Toronto, ON) is a novel point-of-care hyperspectral imaging (HSI) device that allows the acquisition of medical-grade images through a smartphone. HSI acquires a multi-dimensional image dataset (one dimension per imaging modality), called a hypercube, that provides diagnostic information about tissue physiology, morphology, and composition (13). The Ray 1 device is equipped with near- and long-wave infrared sensors, violet light sources, and visible range LEDs, thereby allowing the simultaneous acquisition of visible light, IRT, and BF images as a hypercube. It also integrates into the Swift Skin and Wound app (Swift Medical, Toronto, ON), enabling precise wound area measurement, temperature quantification, and fluorescence area quantification. Under the hypothesis that, through the analysis of the HSI data acquired with the Ray 1 device, wounds can be categorized as not having an associated inflammatory response, having an inflammatory response, or being infected, the objective of this study was to analyze a series of HSI images of patients to determine whether there are differences in the images between infected and non-infected wounds.

## 2. Methods

### 2.1. Patients

**Figure 1:**
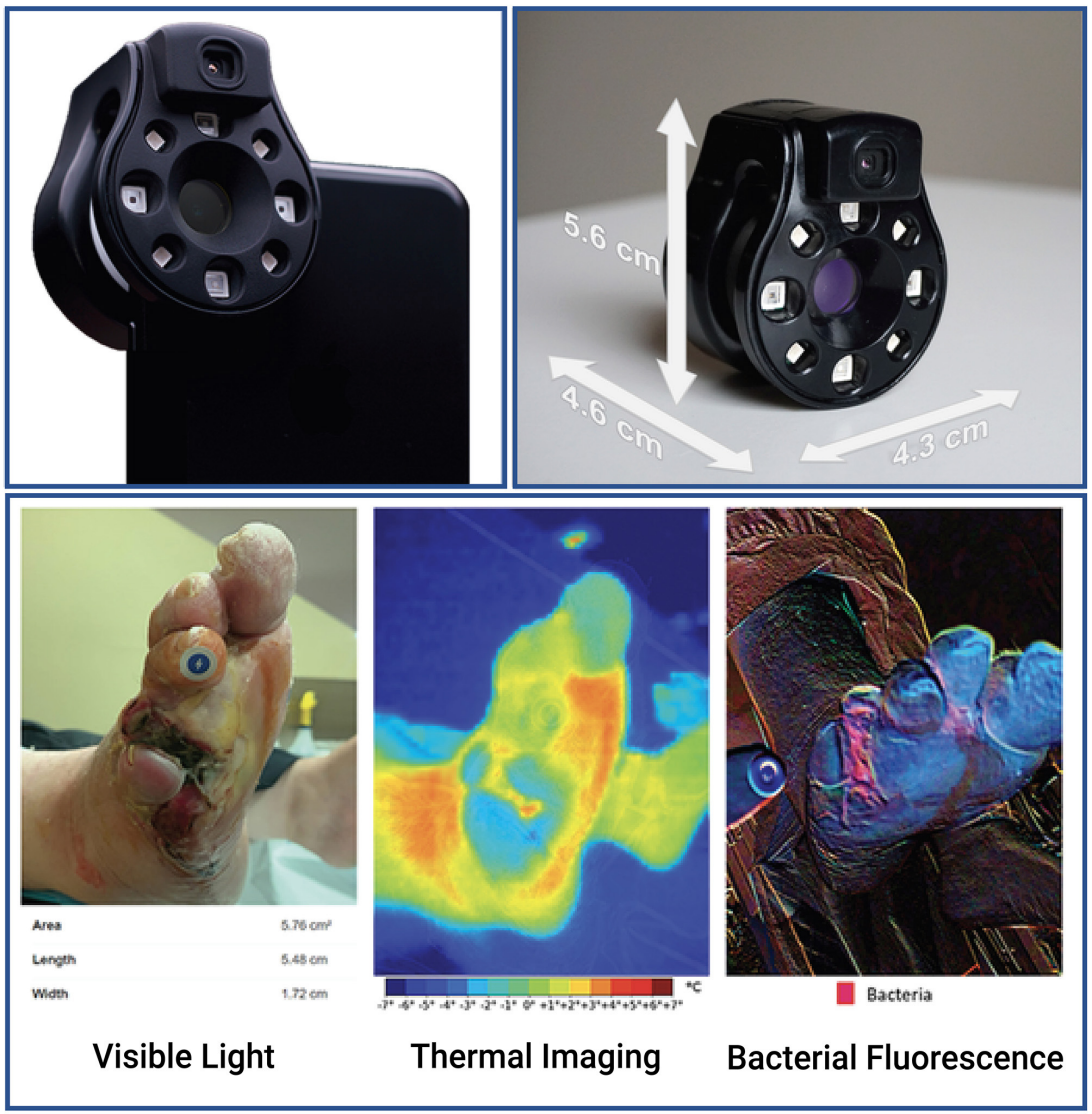
The Ray 1 hyperspectral imaging device. The Ray 1 imaging device is a pocket-sized hyperspectral camera that is designed to sit over a smartphone's camera lens and wirelessly connect to Swift Medical's Skin and Wound app. Once connected, the camera allows the simultaneous acquisition of: visible light images that can be used for clinical inspection, wound area measurement, and the automated identification of tissue types present in the wound; infrared thermal images for the assessment of vascular and inflammatory patterns; and bacterial fluorescence images for the assessment of bacterial bioload in wounds, such as in the vignette presented at the bottom of the figure.

This was a prospective study of patients with either acute or chronic wounds. The patients were enrolled in the Outpatient Wound Clinic of Hospital Central “Dr. Ignacio Morones Prieto” in San Luis Potosí, Mexico or the Foot and Ankle and Orthopedic Clinics of the Montreal General Hospital in Montreal, QC, Canada. The patient images were acquired by trained surgeons using the Swift Skin and Wound app (Swift Medical Inc.), which allows the acquisition and accurate measurement of wound sizes using a fiducial marker (HealX, Swift Medical Inc.), a smartphone (14), and a Ray 1 HSI camera (Swift Medical Inc.) (Figure 1). For this study, a convenience sample was enrolled based on the inclusion criteria to collect visible light images (clinical photographs), thermograms, and bacterial fluorescence images for analysis. The clinical study was designed by and authored by physicians and a nurse (JRGL, MAMJ, RDJF, RB, and GJB) with a minimum of 10 years of clinical experience with wound care. Wound imaging and interpretation were conducted by experienced wound physicians who were trained in the use of the imaging device.

The inclusion criteria for the study were patients over 18 years of age who presented to the clinics with open wounds and consented to participate. The exclusion criteria were patients with wounds with overt signs and symptoms suggestive of extending infection, wounds presenting from foreign objects, or patients previously treated with antibiotics or growth factors. The study was approved by the corresponding Institutional Research Boards (registries 2021/1617 for the Mexican site and 2021-7276 for the Canadian site). All clinical investigations were conducted according to the principles expressed in the Declaration of Helsinki.

### 2.2. Assessment of the infectious status of the wounds

Following the recommendations of the International Wound Infection Institute Wound Infection Continuum (IWII-WIC) (4), wounds were considered infected when an experienced clinician (MAMJ and GKB) considered them to be present with local infection. The IWII-WIC represents the various stages of microbial presence in a wound that increase in severity, from contamination to colonization, local infection, and spreading and systemic infection. Local infection is a stage of infection in which there is the presence and proliferation of microorganisms within the wound that evoke a response from the host. Local infection is contained within the wound and the immediate peri-wound region and is characterized by the presence of hypergranulation, bleeding, friable granulation, epithelial bridging and pocketing, increased exudate, delayed wound healing beyond expectations, erythema, local warmth, swelling, purulent discharge, new or increasing pain, and/or increasing malodor (4). Using these signs and symptoms and the CSS Checklist (6, 15, 16), the wounds were categorized as either clinically infected or non-infected. For patients in the former category, antibiotic treatment was prescribed by their attending physicians as part of the routine care of the wounds.

### 2.3. Imaging protocol

The imaging of the wounds was conducted as follows: first, the wounds were uncovered, cleaned with a saline solution, and patted dry. Loose skin and any blisters present on the skin were removed, and the wounds were allowed to reach room temperature for 5 min. Next, a HealX marker was placed over the unaffected skin adjacent to the wound border, and the Skin and Wound app was used to measure the wound size. Finally, HSI images were acquired at a distance of 15 cm at an angle of 90° relative to the wound bed and with the lights of the examining room on using the Ray 1 imaging device under controlled light, temperature (23°C), and atmosphere humidity settings (40%). For bacterial fluorescence, the images were acquired at 15 cm and 90° relative to the wound bed following the recommendations by Oropallo et al. (17) with slight modifications to comply with the Ray 1 manufacturer's instructions, chiefly to acquire the image under regular light conditions. The Ray 1 device is compatible with a select group of Android and iOS devices, which are available from the manufacturer. Thus, no hooding of the wound or dimming of the lights was required for this imaging modality. For thermal imaging of the wounds, the images were acquired following the TISEM checklist (18) at 15 cm and 90° relative to the wound bed, minimizing the external radiation sources. The skin emissivity was set at 0.98 for all the acquired measurements. After HSI imaging, the wounds were redressed and received standard care.

Image analysis was conducted by a trained researcher blinded to the infectious status of the wounds using Swift Medical's image analysis dashboard. The data extracted included the wound's area in cm^2^, the mean temperature of the wound's bed, the mean temperature of the peri-wound, the mean temperature of a healthy skin control area adjacent to the peri-wound, the temperature difference between the control and wound bed or peri-wound, respectively (thermal asymmetry of the wound and peri-wound), the presence and type (red vs. cyan) of bacterial fluorescence, and the area in cm^2^ of bacterial fluorescence.

### 2.4. Statistical analysis

The data were presented as means, standard deviations, medians, and ranges for continuous parametric or non-parametric data. For categorical variables, the data were presented as proportions. To account for potential variations in clinical characteristics due to the recruitment of patients from two different sites, the linear mixed-effects models were employed to analyze the continuous variables, while the chi-squared tests were conducted for categorical variables. Where appropriate, Tukey's *post-hoc* tests were used for multiple comparison adjustments. The ROC curves were used to identify the optimum cut-off values for assessing the presence of infection vs. no infection for IRT and BF measurements. Data dimensionality reduction was undertaken using principal component analysis (PCA), followed by the k-nearest neighbor clustering (KNN) for identifying groups of patients presenting clinical infection vs. no infection. For creating a PCA-KNN model, the data were split into 80/20% balanced datasets for training and testing. Before testing, the model was 5-fold cross-validated using the test dataset. The model's results reported are those of the test dataset. Statistical analysis was performed on R v.4.0.2 and RStudio v.1.4.17 at 95%CI.

## 3. Results

A total of 66 patients were enrolled in the present study, 16 (24%) from the Montreal General Hospital site and 50 (76%) from Hospital Central “Dr. Ignacio Morones Prieto” in Mexico. The clinical characteristics of the patients are presented in Table 1. A total of 20 wounds (30%) were considered clinically infected. A comparison of the wound's characteristics between the infected and non-infected wounds is presented in Table 2. No significant effect of the patient recruitment site was found for any variable.

**Table 1:** Patient characteristics.

**Variable**	**Value (*n* = 66)**
Age (years)	65 ± 12
Sex	Women = 36 (55%)
	Men = 30 (45%)
Wound type	Diabetic foot ulcer = 27 (41%)
	Pressure ulcer = 16 (24%)
	Trauma = 13 (20%)
	Venous ulcer = 10 (15%)
Wound area (cm^2^)	22 (2–74)

Data are presented as mean ± standard deviation, median and range, or proportions.

**Table 2:** Comparison of infected vs non-infected wounds.

**Variable**	**Non-infected**	**Infected**	***p*-value**
	**(*****n*** = **46)**	**(*****n*** = **20)**	
Age (years)	64 ± 13	67 ± 11	0.29
Sex	Women = 23 (50%)	Women = 13 (65%)	0.38
Wound type	Diabetic foot ulcer = 18 (39%)	Diabetic foot ulcer = 9 (45%)	0.73
	Pressure ulcer = 12 (26%)	Pressure ulcer = 4 (20%)	
	Trauma = 8 (17%)	Trauma = 5 (20%)	
	Venous ulcer = 8 (17%)	Venous ulcer = 2 (15%)	
Wound area (cm^2^)	24.5 (2–72)	21.0 (4–74)	0.80
Wound bed temperature (°C)	29.8 (26.4–34.3)	31.8 (28.1–34.6)	<0.001
Peri-wound temperature (°C)	32.9 (30.9–35.6)	34.8 (31.6–36.8)	0.022
Peri-wound thermal asymmetry (°C)	1.1 (0.3–2.52)	3.0 (1.7–5.3)	<0.001
Positive bacterial fluorescence	13 (28%)	14 (70%)	<0.001
Bacterial fluorescence area (cm^2^)	0.28 (0 to 0.93)	5.35 (0 to 11.9)	<0.001

Data are presented as mean ± standard deviation, median and range, or proportions. Comparisons were made through the linear mixed-effects models or the chi-squared tests or Fisher's exact tests for continuous or categorical data, respectively.

**Figure 2:**
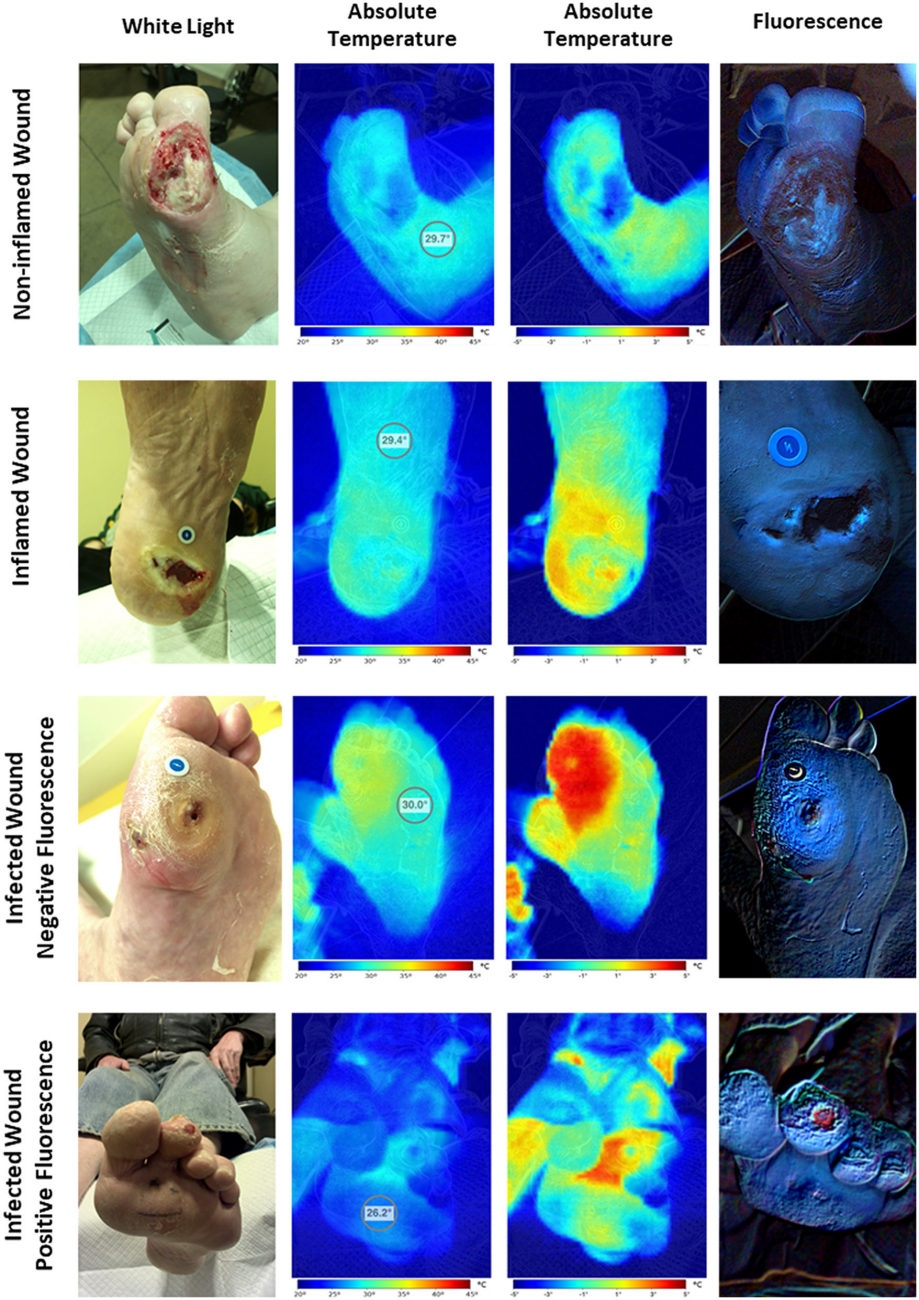
Hyperspectral imaging patterns. The assessment of hyperspectral images of wounds revealed four distinct imaging patterns. The first one corresponds to the non-inflamed wounds characterized by colder wound and peri-wound areas and negative bacterial fluorescence. The second one corresponds to the inflamed wounds characterized by hotter peri-wound areas and cold to moderately warm wound beds with negative to slightly positive bacterial fluorescence. Finally, the third and fourth patterns correspond to the infected wounds characterized by frank hotspots compatible with areas of extensive inflammation with or without positive bacterial fluorescence.

Qualitative analysis of the HSI images showed four distinctive patterns (Table 3, Figure 2). The first pattern identified was wounds with a colder wound bed (decreased wound bed thermal asymmetry, defined as the difference in temperature between the wound bed and a healthy skin control area), a peri-wound thermal asymmetry (defined as the difference in temperature between the peri-wound and a healthy skin control area) <1°C, and a negative signal for bacterial fluorescence. These wounds (*n* = 20, 30%) were considered non-inflamed. The second pattern was characterized by wounds with a decreased to slightly increased thermal asymmetry of the wound bed, those with a moderate increase in the peri-wound thermal asymmetry, and those with a negative to slightly positive bacterial fluorescence. These wounds (*n* = 26, 40%) were considered to be inflamed. The third pattern consisted of wounds with a slightly increased to greatly increased wound bed thermal asymmetry, an increased peri-wound thermal asymmetry, and a negative to slightly positive bacterial fluorescence. Because, in all cases, the attending physician considered these wounds (*n* = 6, 10%) to be infected, we considered them infected but negative for bacterial fluorescence. Finally, the fourth pattern was wounds with a slightly increased to greatly increased wound bed thermal asymmetry, an increased peri-wound thermal asymmetry, and a positive bacterial fluorescence. Notably, all of these wounds were considered to be infected (*n* = 14, 21%). Thus, for the subsequent analyses, we categorized the wounds as non-inflamed, inflamed, or infected.

**Table 3:** Hyperspectral imaging patterns.

**Wound status**	**Wound bed thermal asymmetry**	**Peri-wound thermal asymmetry**	**Bacterial fluorescence**
Non-inflamed	Decreased	Slightly increased	Negative
Inflamed	Decreased to slightly increased	Moderately increased	Negative to slightly positive
Infected with negative fluorescence	Slightly increased to increased	Increased	Negative to slightly positive
Infected with positive fluorescence	Slightly increased to increased	Increased	Positive

**Figure 3:**
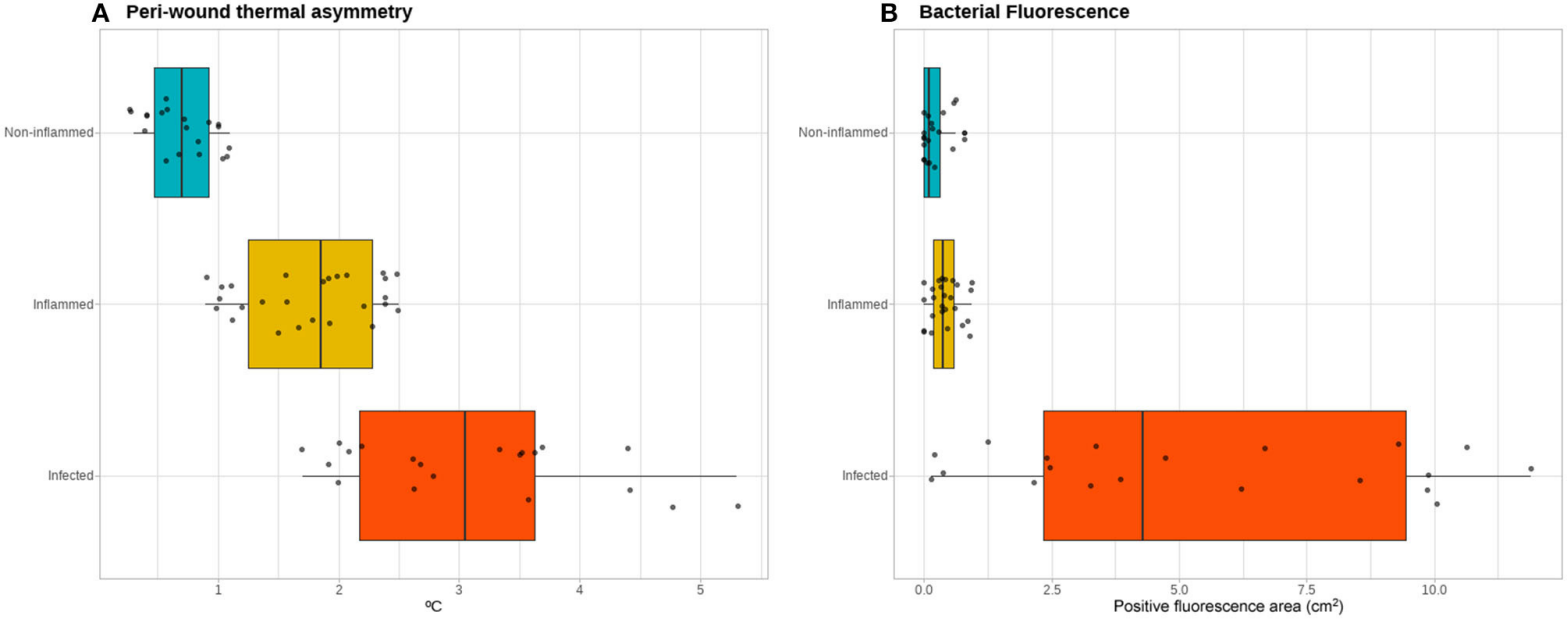
Thermal and bacterial fluorescence quantitative assessment. Quantitative assessment of the infrared thermographic images **(A)** showing a gradient of the severity of the thermal asymmetry of the peri-wound area compared to adjacent healthy skin between the non-inflamed, inflamed, and infected wounds. In contrast, for the quantification of the area positive for bacterial fluorescence **(B)**, the non-inflamed and inflamed wounds do not exhibit a clear gradient, while the infected wounds show a spectrum of positive fluorescence ranging from none detected to over 10 cm^2^ of fluorescence area.

The peri-wound thermal asymmetry between non-inflamed, inflamed, and infected wounds was found to be significant [0.70 (range 0.3 to 1.1) vs. 1.85 (range 0.9 to 2.5) vs. 3.05 (range 1.7 to 5.3)°C, respectively; p <0.001], as well as the fluorescence area [0.09 (range 0 to 0.31) vs. 0.37 (range 0 to 0.93) vs. 3.59 (range 0.73 to 11.90) cm^2^, respectively; *p* <0.001] (Figure 3). A peri-wound thermal asymmetry ≥2.55°C discriminates between the infected and non-infected wounds with a sensitivity of 100%, a specificity of 64%, a positive predictive value of 100%, and a negative predictive value of 72%. For bacterial fluorescence, a positive area of ≥1.65 cm^2^ discriminates between the infected and non-infected wounds with a sensitivity of 100%, a specificity of 55%, a positive predictive value of 100%, and a negative predictive value of 67%.

**Figure 4:**
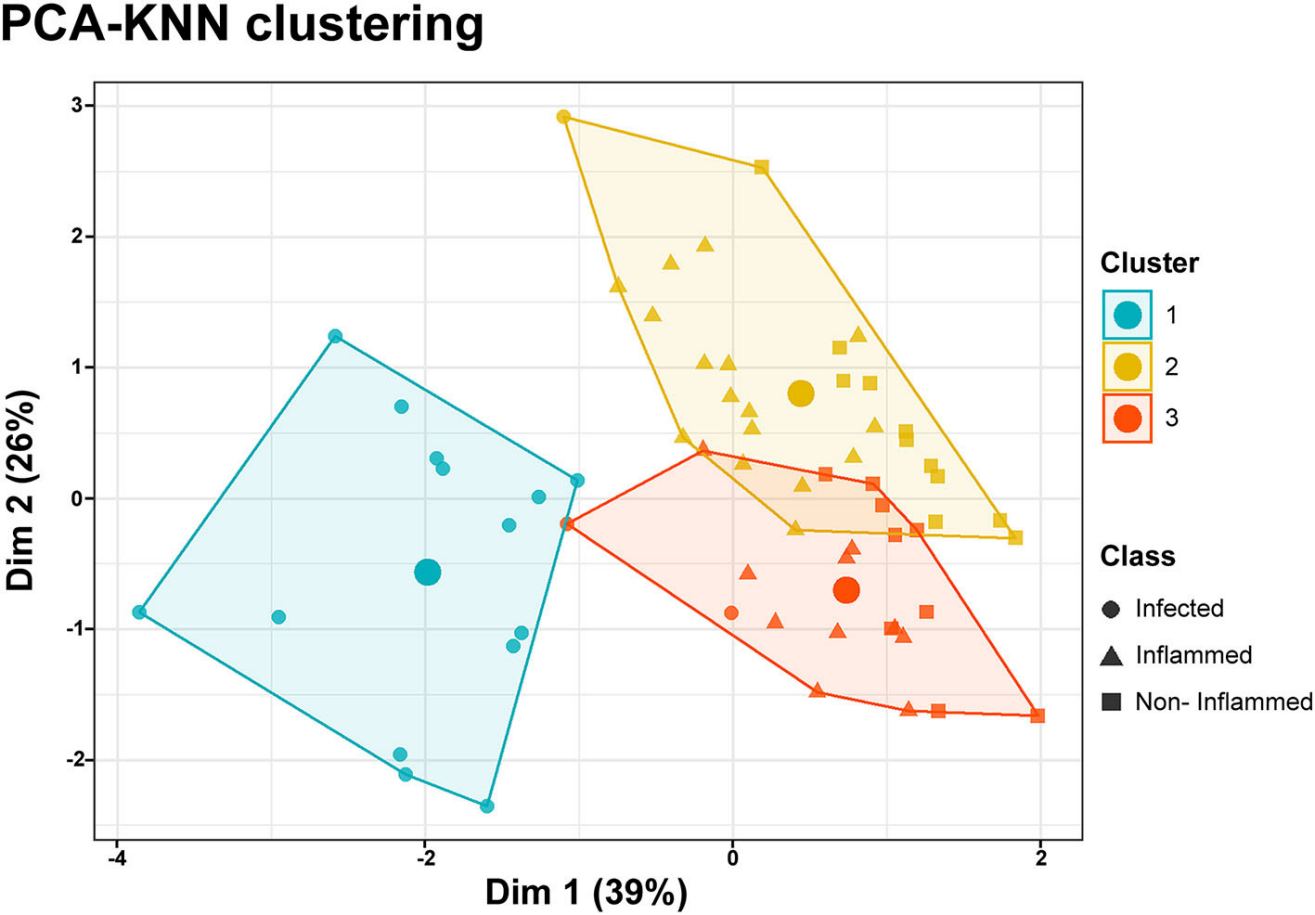
PCA-KNN clustering. Data dimensionality reduction was performed through a principal component analysis (PCA), including clinical and hyperspectral imaging data. K-nearest neighbor (KNN) clustering of the two principal components allows the classification of data points as belonging to three distinct classes encompassing the non-inflamed, inflamed, or infected wounds. The accuracy of the model was 74%. The large circles inside the clusters represent their centroids.

PCA-KNN clustering using all the clinical and HSI variables was able to predict all three wound classes with 74% accuracy. For non-inflamed wounds, the model's sensitivity was 94%, its specificity was 70%, its positive predictive value was 88%, and its negative predictive value was 75%. For inflamed wounds, the model's sensitivity was 85%, its specificity was 77%, its positive predictive value was 85%, and its negative predictive value was 79%. For infected wounds, the model's sensitivity was 100%, its specificity was 91%, its positive predictive value was 100%, and its negative predictive value was 85% (Figure 4).

## 4. Discussion

In recent years, point-of-care IRT and BF imaging have positioned themselves as valuable adjuncts for the assessment of perfusion, inflammation, and infection of wounds (1, 9, 12). However, the widespread adoption of these technologies has been hindered by costs and the requirement to use multiple devices. To our knowledge, although other advanced wound imaging devices are on the market, none combine simultaneous thermography and bacterial fluorescence. Devices appear in different imaging categories, including point-of-care fluorescence imaging (MolecuLight i:XTM, MolecuLight Inc., Toronto, ON, Canada and Illuminate^®^, Adiuvo Diagnostics Private Limited, Chennai, India) and either thermography (FLIR ONE, Teledyne FLIR LLC, Wilsonville, USA), tissue oxygenation (SnapShot IR, Kent Imaging, Calgary, Canada; TIVITA^®^ Mobile, Diaspective Vision GmBH, Salzhausen, Germany), or both (Mimosa Pro, Mimosa Diagnostics, Toronto, Canada). While these advanced imaging technologies alone add value to clinical assessment, combining them may strengthen assessment and diagnostic capabilities, as explored in this pilot study.

The Ray 1 HSI imaging device is the first camera on the market to offer the full range of imaging possibilities that allow the simultaneous acquisition of visible light, with multispectral RGB, IRT, and BF images as a hypercube. Furthermore, this device has the advantages of being pocket-sized and wirelessly connected to a smartphone.

Sandy-Hodgetts et al. (19) found that FL imaging was more accurate than the CSS alone in predicting high levels (>10^4^ CFU). However, bacterial presence does not determine infection in wounds. They found that FL imaging had moderate sensitivity and specificity for identifying wounds with bacteria, and CSS had very low sensitivity but high specificity. The results of our study indicate that adding thermography may help advance the screening potential of bacterial presence in wounds to correctly identify infection. A cross-sectional study by Woo et al. (20) assessed a CSS screening tool and found that temperature had the most significant predictive value for identifying infection (odds ratio of 8.05, sensitivity 76%, specificity 71%).

Thermography adds an objective approach to assessing temperature patterns in wound assessment. Derwin et al. (21) collected thermographic and fluorescence imaging data for a feasibility study, monitoring the temperatures of 26 wound patients. Although the study captured separate advanced images (fluorescence and thermography), the authors did not screen for or identify infection as an outcome. The study monitored wound temperature readings at the wound center, the hottest temperatures at the wound edge and within the wound bed, and average wound bed temperature readings. In contrast, our study identified patterns in temperature gradients to monitor the body's response to the wound and screen for signs of potential infection. Specifically, we looked for temperature asymmetry as areas of increased temperature in the wound base and peri-wound may result from inflammatory processes. Absolute temperature alone may be challenging to use for infection detection. Derwin et al. (21) noted that skin temperature varies based on body locations and other factors (e.g., environmental, circulatory), so temperature differences and gradients may prove more effective than absolute temperature alone for infection detection.

In this initial report on the use of HSI imaging to assess the infectious status of a wound, we have demonstrated that the combined use of patient clinical data, visible light, IRT, and BF imaging can be used to discriminate between infected vs. non-infected wounds and to further categorize them as being non-inflamed, inflamed, or infected with an accuracy of 74%. Interestingly, while the clinical characteristics of wounds showed no significant differences between the infected and non-infected groups (Table 2), the HSI data show significant differences among them. Thus, it can safely be assumed that the main drivers for the PCA-KNN algorithm are the IRT and BF measurements. While each one of these imaging modalities has made successful penetration into wound care, as standalone applications, they suffer from relatively high sensitivity but low specificity, which results in significant numbers of false positives (22–25). In the cases of IRT and BF, the false positive would be an inflammation without infection or bacterial colonization, respectively. As our results show, combining the imaging modalities makes it possible to significantly increase their overall accuracy in detecting infection by augmenting their specificity almost twofold while maintaining 100% sensitivity. The main differences identified between the non-infected and infected groups are increases in the gradient of the thermal asymmetry between the peri-wound area and an adjacent healthy skin area and the area of positive bacterial fluorescence, which cannot be detected by visual or clinical inspection alone. These features highlight the notion that HSI imaging can be used for “below-the-skin” diagnostics and that these images offer powerful insights into wound healing.

In the current standard of care, whenever a patient's wound is suspected to be infected, after an initial clinical evaluation that should include the assessment of the wound and peri-wound regions for erythema, pain, discharge, tissue changes, and the presence of malodor, the ensuing diagnostic testing includes the identification of inflammatory serum markers such as leukocytes, platelets, c-reactive protein, and a microbiological assessment of the wound bed. The latter should be done through the Levine technique of swabbing or tissue biopsy, as it is widely accepted that any swabbing of the tissue bed mostly represents contamination of the wound and not the infectious process itself (4, 26). Unfortunately, microbiological analysis of wound swabs can only identify microorganisms on the surface of a wound or at the depth of the tissue biopsy taken; thus, deeper pockets of infection can be missed on these assessments (27). Furthermore, it should be noted that, while laboratory and microbiological assessments are objective, these assessments may miss local changes that have not yet elicited a systemic inflammatory response in the former case and suffer from the operator's experience in acquiring the sample in the former (28). Thus, these conditions represent significant gaps that can be filled with the concurrent use of HSI imaging.

It is noteworthy to mention that the presence of bacteria in the wound bed or the peri-wound area does not equal infection. As demonstrated in our study, both non-inflamed and inflamed wounds may show areas of BF. However, the timely identification of bacterial colonization is critical for optimizing wound care, as bacterial contaminants, even in moderate amounts, are known to delay healing and render the wound bed unable to receive advanced therapies, including skin grafts and cellular-based products (29). Moreover, once a wound has been identified as “hot” and inflamed and with areas positive for bacterial fluorescence, care must be taken to ensure this condition does not reflect an incipient infection, and close observation is warranted.

In contrast, “cold” wounds, identified in the present study as non-inflamed, likely reflect tissue with low healing potential due to a limited blood supply. Previous research has demonstrated that temperature measurements closely correlate with the presence of blood flow and blood vessel density (30, 31) and that the thermal asymmetry between healthy skin and a wound's bed is predictive of the time required for healing (32) and the treatment it will require for achieving closure (31). However, more research is needed to sustain this hypothesis.

Per the current interpretation guidelines (12), BF should always be compared to standard clinical photographs to provide a clear anatomical context of the signals observed, and the same recommendation can be extended to IRT imaging. Brighter areas of fluorescence may occur with more superficial areas of bacteria. Therefore, brighter areas may not necessarily indicate a worse wound prognosis if bacteria can be addressed by adequate debridement. Faint signals may occur when there is a high level of bacteria underneath the surface, which may be more clinically significant, which is why BF imaging guideline advancement may be significantly enhanced by the integration of other advanced imaging modalities, such as thermography.

The device used in this study is the only one on the market currently supporting the concomitant acquisition of images in the visible light and BF ranges. In addition, it also acquires simultaneous IRT images. Thus, it is the only imaging device that enables the precise anatomical co-localization of the different signals of the hypercube. In our opinion, this represents the greatest strength of our research, as this feature has never been explored before. Limitations to the research include a lack of systematic, objective infection measurements, such as tissue biopsies, as the classification of infected vs. non-infected wounds was clinically done.

Future studies are planned to address this pitfall and assess whether the use of targeted tissue biopsies increases the diagnostic yield of imaging adjuncts. This pilot study suggests that combining simultaneous imaging modalities may provide clinicians with a more objective infection screening tool. To validate these findings, further studies that are adequately powered with more quantitative infection measures are necessary. Thermography and fluorescence image interpretation would benefit from investigations into their various patterns. A meta-analysis of thermography's role in medical imaging, such as burn depth or diabetic foot complications, concluded that machine learning shows positive signs in image interpretation (33).

Further investigation is required to determine if the thermographic and bacterial fluorescence patterns may emerge based on the pathogens or degree of infection. Using Artificial Intelligence to detect these patterns may improve the reliability of these technologies. Previous studies noted a variance in sensitivity and specificity between experts and non-experts (19). Incorporating AI-based diagnostic and prognostic indices could increase the utility of advanced wound imaging and support wider adoption. Integrating machine learning techniques to add objective feature extraction, such as wound segmentation and tissue type quantification (34), regions of interest quantification in thermographic and bacterial fluoresce images, or objective wound healing risk prediction (35) based on integrated advanced wound imaging, are important areas for investigation. The technology features must be evaluated with a focus on more comprehensive wound assessment, reducing clinical training requirements and enabling better clinician workflows.

## 5. Conclusion

In conclusion, the combination of visible light wound imaging, IRT, and BF increases the sensitivity and specificity of infection detection and helps categorize non-infected wounds as inflamed or non-inflamed. This categorization can then be used to provide a more rational and targeted treatment, assess the causes of non-healing as perfusion-based or infection-based, evaluate the need for changing the type of wound dressings, and monitor the response to treatments. Therefore, the advent of this pocket-size HSI imaging device capable of offering this information as an “all-in-one” device holds great promise for enabling the point-of-care assessment of perfusion, inflammation, and infection.

## Data availability statement

The datasets presented in this study can be found in online repositories. The names of the repository/repositories and accession number(s) can be found below: https://borealisdata.ca/dataverse/jrg_experimental_surgery.

## Device specifications

The Ray 1 (Swift Medical Inc., Toronto, Canada) device used in this study had a thermal camera with a resolution of 80 x60 and multi-point high-powered LEDs using pulse-width modulation for intensity control for bacterial fluorescence imaging.

## Ethics statement

The studies involving human participants were reviewed and approved by Hospital Central Dr. Ignacio Morones Prieto, San Luis Potosí, Mexico–Study# 2021/1617 2, McGill University Health Centre, Montreal, QC, Canada–Study #2021-7276. The patients/participants provided their written informed consent to participate in this study.

## Author contributions

JR-G: conceptualization, data collection, statistical analysis, writing, and editing. MM-J and RF: data collection, writing, and editing. RB, AL, GS, and GB: writing and editing. ZL: software development, writing, and editing. All authors contributed to the article and approved the submitted version.
